# Molecular hydrogen and osteoarthritis: multi-target mechanisms and therapeutic effectiveness

**DOI:** 10.4103/mgr.MEDGASRES-D-25-00332

**Published:** 2026-09-12

**Authors:** Jiayu Chen, Dong Pan, Yixiang Zhao, Junyu Lin, Jie Tong

**Affiliations:** 1 Department of Spinal Surgery, The First People’s Hospital of Chenzhou, Chenzhou, Hunan Province, China; 2 Translational Medicine Institute, the First People’s Hospital of Chenzhou Affiliated to University of South China, Chenzhou, Hunan Province, China; 3 Department of Orthopedics, The Second Hospital of Lanzhou University, Lanzhou, Gansu Province, China; 4 Division of Joint Surgery, Department of Orthopedics, Guangzhou First People’s Hospital, School of Medicine, South China University of Technology, Guangzhou, Guangdong Province, China

**Keywords:** anti-inflammatory, chondroprotection, clinical translation, disease-modifying osteoarthritis drug, drug delivery system, mitophagy, molecular hydrogen, osteoarthritis, oxidative stress, pharmacokinetics

## Abstract

Osteoarthritis is a prevalent degenerative joint disease characterized by a self-perpetuating cycle of chronic inflammation, oxidative stress, and extracellular matrix degradation. Current therapies provide only symptomatic relief and do not alter disease progression, highlighting the urgent need for effective disease-modifying osteoarthritis drugs. Molecular hydrogen has emerged as a promising therapeutic candidate due to its unique selective antioxidant, anti-inflammatory, and anti-apoptotic properties. This review summarizes the multi-target mechanisms of molecular hydrogen and highlights innovative delivery technologies designed to overcome its key pharmacokinetic limitations, especially rapid diffusion and short residence time in the joint. We critically evaluate advanced strategies ranging from sustained-release injectable depots to stimulus-responsive nanocarriers and cell-based delivery systems. Clinical translation remains at an early stage; progress will depend on integrating intelligent, localized delivery systems with rigorously designed clinical trials to validate molecular hydrogen as a safe and effective disease-modifying osteoarthritis drug.

## Introduction

Osteoarthritis (OA) is the most common degenerative joint disease worldwide, characterized by progressive articular cartilage degradation, osteophyte formation, and synovial inflammation. It has become a major global public health concern.^1^ Epidemiological studies estimate that OA affects more than 595 million people worldwide, and its prevalence and burden are rising with population aging and increasing obesity.^2^,^3^ This condition not only causes chronic pain and functional disability in patients but also imposes a substantial socioeconomic burden due to escalating healthcare costs.^1^,^3^,^4^

The pathogenesis of OA extends far beyond simple “mechanical wear and tear.” Instead, OA is a complex, whole-joint disease driven by the interplay of chronic low-grade inflammation and oxidative stress.^1^,^5^ At the molecular level, pro-inflammatory cytokines, such as interleukin-1β and tumor necrosis factor-α, continuously activate signaling cascades, including nuclear factor-κB (NF-κB).^6^,^7^ This activation upregulates matrix-degrading enzymes, such as matrix metalloproteinase-13, in chondrocytes. The resulting catabolic shift accelerates extracellular matrix (ECM) breakdown and cartilage degeneration.^5^,^8^,^9^,^10^,^11^ Meanwhile, excessive reactive oxygen species (ROS) generation within the joint directly injures chondrocytes. It also amplifies inflammatory signaling and promotes chondrocyte apoptosis and senescence.^9^,^12^ Senescent cells also release a senescence-associated secretory phenotype rich in pro-inflammatory and matrix-degrading factors, which further worsen oxidative stress and inflammation in the joint microenvironment.^13^ Overall, OA is a multifactorial, self-amplifying network involving inflammation, oxidative stress, apoptosis, and cellular senescence.^9^,^10^,^12^,^13^

Despite extensive research efforts, current clinical management of OA largely focuses on symptomatic relief rather than true disease modification.^14^,^15^,^16^ First-line pharmacological treatments, such as nonsteroidal anti-inflammatory drugs, provide temporary alleviation of pain and inflammation but are often accompanied by significant adverse effects, including gastrointestinal and cardiovascular complications.^16^,^17^ Intra-articular injections, such as corticosteroids or hyaluronic acid, may offer short-term symptom relief but do not prevent or reverse structural deterioration.^18^ Consequently, many patients ultimately require total joint replacement surgery, which is invasive, costly, and carries the risk of postoperative complications.^16^,^17^

More importantly, multiple candidate disease-modifying OA drugs (DMOADs) have failed in clinical trials despite a strong rationale. Examples include the interleukin-1 receptor antagonist anakinra and matrix metalloproteinase inhibitors.^19^,^20^ These setbacks indicate a significant therapeutic gap in OA management and suggest the urgent need for novel, safe, and multi-targeted therapeutic strategies.^15^

Given this multifaceted pathogenesis, an ideal therapeutic agent would need to concurrently address oxidative stress, inflammation, and matrix degradation without disrupting essential physiological processes.^5^,^8^,^9^ Against this background, molecular hydrogen (H_2_) has emerged as a promising medical gas with unique therapeutic potential.^21^,^22^ A distinctive feature of H_2_ is its selective antioxidant activity: it scavenges highly cytotoxic species, especially hydroxyl radicals (·OH), while largely sparing physiologically required redox signaling.^23^ Furthermore, H_2_ exerts multi-target anti-inflammatory and anti-apoptotic effects through the suppression of key signaling pathways, such as NF-κB and related cascades.^6^,^7^,^21^ Due to its extremely small molecular size, high diffusibility, and excellent biosafety profile, H_2_ is an innovative and physiologically compatible approach for targeting the core pathological processes of OA.^21^,^22^,^24^

Despite accumulating preclinical evidence, clinical translation is hindered by two linked gaps: an incomplete, unified account of H_2_ mechanisms and major pharmacokinetic challenges in generating and delivering H_2_ to the joint.^22^,^24^,^25^,^26^ Therefore, this review aims to (1) synthesize how H_2_ disrupts the OA pathological network through multi-target mechanisms and (2) critically evaluate how its central limitation, a transient pharmacokinetic profile, is being addressed by next-generation intelligent delivery platforms. We argue that integrating mechanistic understanding with advanced delivery engineering is key to unlocking the clinical potential of H_2_ as a DMOAD. **Figure 1** provides a graphical overview of the pathological network of OA, the major therapeutic actions of H_2_, and delivery strategies designed to optimize joint exposure. Collectively, this work seeks to provide a solid theoretical and translational framework for developing molecular H_2_ as a novel class of DMOADs.

**Figure 1:**
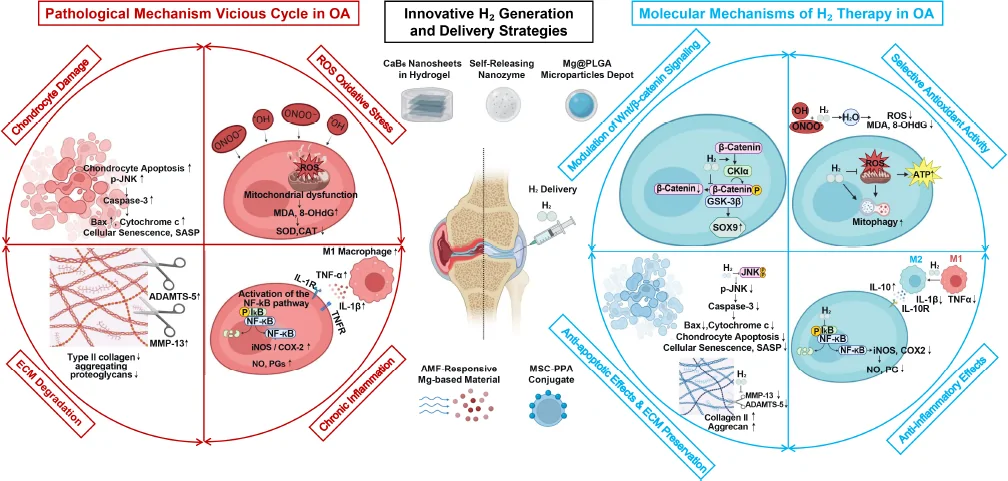
H_2_ as an exposure-limited, multi-target intervention in osteoarthritis (OA), enabled by engineered delivery strategies. ·OH: Hydroxyl radical; 8-OHdG: 8-hydroxy-2′-deoxyguanosine; ADAMTS: a disintegrin and metalloproteinase with thrombospondin motifs; AMF: alternating magnetic field; ATP: adenosine triphosphate; Bax: Bcl-2-associated X protein; CAT: catalase; CK1: casein kinase 1; COX-2: cyclooxygenase 2; ECM: extracellular matrix; GSK3β: glycogen synthase kinase 3β; H_2_: molecular hydrogen; iNOS: inducible nitric oxide synthase; IκB: inhibitor of NF-κB; JNK: c-Jun N-terminal kinase; MDA: malondialdehyde; MMP: matrix metalloproteinase; NF-κB: nuclear factor-κB; NO: nitric oxide; ONOO^−^: peroxynitrite; PGs: prostaglandins; PPA: porphyrin-polyphenol-albumin; ROS: reactive oxygen species; SASP: senescence-associated secretory phenotype; SOD: superoxide dismutase; SOX9: SRY-box transcription factor 9.

To facilitate this evidence-to-translation narrative, this review synthesizes mechanistic and pharmacological foundations with existing preclinical and clinical data, and proposes an exposure-optimized development roadmap for H_2_ as a potential disease-modifying therapy for OA.

## Search Strategy

To evaluate the evidence for H_2_ in OA, we conducted a systematic search for primary studies published between January 2005 and October 2025. Using a predefined screening process (**Additional file 1**), we identified 10 core studies. These include nine preclinical studies and one clinical trial directly investigating H_2_ therapy in OA models or patients. These studies^6^,^27^,^28^,^29^,^30^,^31^,^32^,^33^,^34^,^35^ constitute the primary OA-specific evidence base for H_2_ therapy and therefore form the foundation of our mechanistic and translational analysis (**Table 1**), including consistent outcome domains and key limitations such as exposure-limited pharmacokinetics. Additional references cited for context (e.g., OA pathophysiology and general delivery technologies) were not included in this core evidence synthesis.

**Table 1:** Experimental studies and clinical trial of H_2_ in OA

Study	Year	Study design	H_2_ delivery method	Results
**Experimental study**				
Hanaoka et al.^27^	2011	*In vitro*: Porcine/rat chondrocytes (stimulated by SNAP)	H_2_ (dissolved in culture medium)	Protects chondrocytes by reducing peroxynitrite (ONOO−). Restores matrix gene expression (↑Aggrecan, Col II; ↓MMP3, MMP13).
Cai et al.^6^	2013	*In vitro*: Neonatal rat osteoblasts (stimulated by TNF-α)	H_2_ (hydrogen-rich medium)	Alleviates cell injury by abating oxidative stress, preserving mitochondrial function, and suppressing NF-κB pathway activation and inflammation.
Lin et al.^28^	2016	*In vitro*: Human OA chondrocytes; *In vivo*: Rat surgery-induced OA model	Cell gas exposure or oral H_2_ water	Suppresses abnormally activated Wnt/β-catenin signaling by promoting β-catenin degradation.
Wan et al.^29^	^2018^	*In vivo*: Mouse OA model	Intramuscular depot (Mg@PLGA microparticles)	Provides continuous H_2_ evolution in situ, effectively mitigating tissue inflammation and preventing cartilage destruction.
Cheng et al.^30^	2020	*In vivo*: Rat OA model (modified Hulth method)	Intra-articular HW	Attenuates cartilage degradation via anti-oxidant, anti-apoptotic, and anti-catabolic effects (↓MMP3, MMP13, ADAMTS4, ADAMTS5; ↓COX-2, iNOS).
Lu et al.^31^	2021	*In vitro*: Human chondrocytes (TBHP-induced); *In vivo*: Mouse DMM model	Cell H_2_ gas exposure or animal H_2_ inhalation	Alleviates apoptosis and ECM degradation (↓ADAMTS5, MMP13, cleaved caspase-3) by inhibiting the JNK signaling pathway.
Zhang et al.^32^	2023	*In vitro*: Macrophages, chondrocytes; *In vivo*: Rat OA model	Injectable H_2_-releasing hydrogel (CBN@GelDA)	Provides sustained H_2_ release; scavenges ROS, reduces inflammation (M1→M2 macrophage polarization), and decreases chondrocyte apoptosis.
Xu et al.^33^	2025	*In vivo*: Severe OA model	H_2_ generators-backpacked MSCs (systemic transplant)	H_2_ remodels articular redox balance, preserves MSC function, reverses immune dysfunction (M2 polarization), and promotes cartilage regeneration.
Wang et al.^34^	2025	*In vitro*/*In vivo*: Chondrocytes/OA model	H_2_-releasing nanozymes (Se-HMPB@AB@COS)	Reduces oxidative stress (↓·OH), reverses mitochondrial dysfunction, enhances mitophagy, and delays chondrocyte senescence.
**Clinical trial**				
Wang et al.^35^	2025	Open-label, blinded-endpoint, RCT; Population: 121 elderly KOA patients (avg. age 81.2)	H_2_–O_2_ inhalation (60 min/d, 2 wk) + HBE *vs.* HBE alone	H_2_–O_2_ inhalation alleviated KOA symptoms and enhanced functional activity during the initial 2 wk. However, the effects were not sustained at 12 wk, and no significant difference was observed compared to the control group (*P* = 0.140).

↑: Increased/upregulated; ↓: decreased/downregulated; ·OH: hydroxyl radical; ADAMTS: a disintegrin and metalloproteinase with thrombospondin motifs; avg: average; CBN@GelDA: calcium boride nanosheet-loaded gelatin methacryloyl hydrogel; Col II: type II collagen; COX-2: cyclooxygenase 2; DMM: destabilization of the medial meniscus; ECM: extracellular matrix; H_2_: molecular hydrogen; H_2_–O_2_: hydrogen–oxygen inhalation; HBE: home-based exercise; HW: hydrogen-rich water; iNOS: inducible nitric oxide synthase; JNK: c-Jun N-terminal kinase; KOA: knee osteoarthritis; Mg@PLGA: magnesium powder-encapsulated poly(lactic-co-glycolic acid) microparticles; MMP: matrix metalloproteinase; MSC: mesenchymal stem cell; NF-κB: nuclear factor-κB; OA: osteoarthritis; ONOO^−^: peroxynitrite; RCT: randomized controlled trial; ROS: reactive oxygen species; Se-HMPB@AB@COS: hydrogen-releasing nanozymes; SNAP: S-nitroso-N-acetylpenicillamine; TBHP: tert-butyl hydroperoxide; TNF-α: tumor necrosis factor alpha.

## Molecular Mechanisms of Hydrogen Therapy in Osteoarthritis

Given the complex pathological network underlying OA, therapeutic strategies targeting a single molecular pathway often yield limited efficacy.^1^,^10^ H_2_, with its favorable safety profile and a unique combination of antioxidant, anti-inflammatory, and cytoprotective properties, is an attractive candidate to address this complexity within the OA pathological network.^21^,^23^,^30^,^31^,^34^ These convergent actions support a multi-target model in which H_2_ modulates redox balance, inflammatory signaling, cell-fate programs, and matrix homeostasis in OA (**Figure 2**).

**Figure 2:**
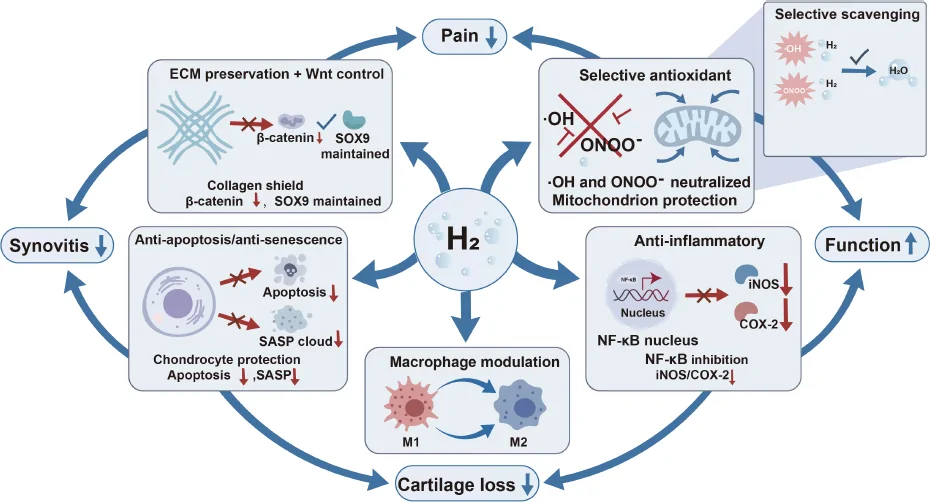
Multi-target therapeutic mechanisms proposed for H_2_ in OA. COX-2: Cyclooxygenase 2; ECM: extracellular matrix; H_2_: molecular hydrogen; iNOS: inducible nitric oxide synthase; NF-κB: nuclear factor-κB; OA: osteoarthritis; ONOO^−^: peroxynitrite; SASP: senescence-associated secretory phenotype; Wnt: wingless/integrated signaling.

### Inhibition of oxidative stress

Oxidative stress is a pivotal driver of the self-perpetuating OA pathology, directly damaging chondrocytes and amplifying inflammatory and catabolic signaling.^9^,^10^,^23^ H_2_ counters this via selective antioxidant activity, with accumulating evidence supporting efficient neutralization of highly cytotoxic radicals, particularly ·OH and peroxynitrite (ONOO^−^).^9^,^23^,^27^ In OA models, this primary action is reflected by reduced oxidative injury markers, including protein nitration, lipid peroxidation (e.g., malondialdehyde), and DNA damage (e.g., 8-hydroxy-2′-deoxyguanosine).^30^,^31^,^32^ Together, these changes help preserve chondrocyte structure and function under stress.

The protective effects of H_2_ extend to the organelle level. Because of its small size, H_2_ can diffuse into mitochondria, where it helps stabilize membrane potential, support adenosine triphosphate production, and promote mitophagy (selective clearance of damaged mitochondria). By preserving mitochondrial integrity and enhancing mitochondrial quality control, H_2_ addresses a major source of pathological ROS within chondrocytes.^12^,^34^ H_2_ treatment is also associated with upregulation of endogenous antioxidant enzymes, such as superoxide dismutase and catalase, suggesting that it can strengthen redox homeostasis rather than acting only as a direct scavenger.^21^,^30^,^32^

In summary, H_2_ acts as a multi-level redox regulator in OA by directly quenching cytotoxic radicals, preserving mitochondrial function, and bolstering endogenous defenses. By attenuating oxidative stress, an upstream amplifier of the OA vicious cycle, H_2_ creates conditions that favor downstream anti-inflammatory and anti-apoptotic effects.^21^,^31^

### Anti-inflammatory effects and extracellular matrix protection

The anti-inflammatory effects of molecular H_2_ are multifaceted and play a central role in mitigating the progression of OA. Primarily, H_2_ suppresses classical inflammatory signaling by inhibiting NF-κB activation, which downregulates inducible nitric oxide synthase and cyclooxygenase-2 expression and reduces production of inflammatory mediators such as nitric oxide and prostaglandins.^6^,^7^,^27^,^30^,^31^,^33^,^36^

In addition to directly suppressing inflammatory cascades, H_2_ modulates the intra-articular immune microenvironment.^33^ H_2_ promotes macrophage polarization from the pro-inflammatory M1 phenotype to the anti-inflammatory M2 phenotype, reducing pro-inflammatory cytokine release and dampening chronic inflammatory reinforcement in OA.^33^,^37^ Furthermore, H_2_ acts synergistically with the paracrine effects of mesenchymal stem cells (MSCs) to enhance M2 macrophage polarization and restore immune homeostasis within the joint.^33^

Ultimately, the combined anti-inflammatory and antioxidative activities of H_2_ contribute to the preservation of the ECM.^29^,^30^,^32^ H_2_ downregulates key matrix-degrading enzymes, including matrix metalloproteinase-3, matrix metalloproteinase-13, and a disintegrin and metalloproteinase with thrombospondin motifs 4/5 (ADAMTS-4/5), thereby slowing ECM breakdown.^13^,^30^,^32^ In parallel, it supports anabolic markers such as type II collagen and aggrecan.^27^,^29^,^30^,^31^

### Regulation of apoptosis and redox-sensitive signaling networks

Chondrocyte apoptosis is a hallmark of cartilage degeneration in OA.^10^,^12^,^13^ Across preclinical models, H_2_ shows a consistent dual benefit: preserving chondrocyte viability while maintaining ECM homeostasis.^29^,^30^,^31^,^32^,^34^ This is not merely a parallel observation but a mechanistically coupled effect, reflecting shared redox-sensitive control of survival and matrix homeostasis.^9^,^22^ Mechanistically, H_2_ functions by interrupting the crosstalk between redox imbalance and stress-activated protein kinases.^9^,^31^

A central transmission node is the stress-kinase axis, especially c-Jun N-terminal kinase (JNK).^31^ Under oxidative or inflammatory stress, JNK activation promotes mitochondrial apoptosis (e.g., Bax activation, cytochrome c release, and caspase-3 cleavage) and can also reinforce catabolic transcription.^5^,^31^,^38^ In stressed chondrocyte models, H_2_ treatment is associated with reduced JNK phosphorylation, fewer apoptotic cells, and lower expression of key catabolic enzymes such as matrix metalloproteinase-13 and ADAMTS5. Critically, where mechanistic perturbations are performed, pharmacological reactivation of JNK diminishes the chondroprotective efficacy of H_2_, supporting JNK as a critical redox-sensitive mediator. These *in vitro* observations are corroborated *in vivo*. H_2_ administration in surgically induced OA models correlates with reduced levels of p-JNK in cartilage, improved proteoglycan retention, and better histological scores.^31^

Beyond the stress-kinase axis, H_2_ also modulates the Wnt/β-catenin signaling pathway, which is often aberrantly reactivated in OA.^11^,^28^ H_2_ has been reported to facilitate β-catenin phosphorylation and proteasome-directed turnover via the canonical destruction complex, thereby limiting pathological β-catenin accumulation.^28^ By limiting abnormal nuclear β-catenin accumulation and reducing transcription of downstream targets, H_2_ helps preserve chondrocyte identity and is accompanied by sustained expression of the master transcription factor SOX9.^11^,^28^

Overall, current evidence supports a “network modulation” model in which H_2_ reduces redox stress and, as a result, dampens multiple stress-responsive pathways converging on apoptosis control and ECM homeostasis rather than acting as a single-pathway inhibitor.^22^,^31^,^34^

### Beyond apoptosis: autophagy and emerging regulated cell death modalities

OA progression involves more than apoptosis; it also includes autophagy dysfunction, ferroptosis, and pyroptosis, all of which are closely linked to pathological redox imbalance.^9^,^39^,^40^,^41^,^42^ This section prioritizes autophagy, for which direct experimental evidence exists in OA models.^42^ Subsequently, we examine ferroptosis and pyroptosis as emerging therapeutic targets; while direct validation is evolving, their redox-dependent nature implies a high therapeutic potential for H_2_.^21^,^23^,^39^,^40^,^41^,^43^

#### Autophagy and mitophagy: restoring cellular quality control

Autophagy maintains chondrocyte homeostasis by clearing damaged proteins and organelles; mitophagy selectively removes dysfunctional mitochondria.^42^ OA is associated with impaired autophagic flux, which exacerbates mitochondrial dysfunction and inflammatory signaling.^12^,^42^ Direct evidence from OA models indicates that H_2_-based interventions can enhance mitochondrial quality control. For example, a spontaneously H_2_-releasing nanoenzyme (Se-HMPB@AB@COS) was reported to reduce ROS while also enhancing mitophagy and promoting ECM synthesis.^34^ This suggests that H_2_ can reactivate the chondrocyte’s intrinsic repair machinery, connecting its redox action to durable cellular resilience.^22^,^34^

#### Ferroptosis: a plausible target via redox regulation

Ferroptosis is an iron-dependent regulated cell death driven by lethal lipid peroxidation.^44^ While increasingly implicated in OA cartilage injury, direct evidence that H_2_ suppresses ferroptosis in OA remains limited.^40^,^41^ The mechanistic rationale, however, is strong. H_2_ is a well-established inhibitor of lipid peroxidation.^23^,^45^ In non-OA models (e.g., cardiac injury), H_2_ treatment attenuates ferroptosis, an effect linked to nuclear factor erythroid 2-related factor 2 (Nrf2) pathway activation.^46^ To validate the role of ferroptosis in this context, future studies should determine whether H_2_ reduces lipid peroxidation, preserves glutathione peroxidase 4 (GPX4)/glutathione defenses, and mitigates chondrocyte loss under ferroptosis-provoking conditions.^40^,^41^

#### Pyroptosis: dampening the inflammatory burst

Pyroptosis is an inflammasome-linked, lytic form of cell death that releases interleukin-1β/interleukin-18, contributing to OA synovitis. Specific studies on H_2_-mediated inhibition of pyroptosis in OA are scarce.^39^,^47^ However, in other inflammatory conditions, H_2_ has been reported to suppress the NOD-like receptor thermal protein domain associated protein 3 (NLRP3) inflammasome activation.^43^ Future research should test whether H_2_ inhibits inflammasome activation and gasdermin cleavage in synovial and cartilage tissues and whether this interrupts inflammation-driven cell death.^39^,^48^

### Molecular targets and a hierarchical mechanistic framework

The broad spectrum of therapeutic effects detailed above raises a fundamental pharmacological question: What is the primary molecular target of H_2_? Unlike conventional small-molecule drugs that function via “lock-and-key” binding to specific protein pockets, H_2_ appears to operate through a distinct mechanism.^21^,^22^,^23^,^27^ Current evidence suggests that the direct targets of H_2_ are not proteins, but highly cytotoxic reactive oxygen/nitrogen species, specifically the ·OH and ONOO^−^. By selectively neutralizing these radicals, H_2_ functions effectively as a biological “Redox Buffer.”^21^,^23^

This distinction is crucial for interpreting its mechanism. These downstream pathway changes, such as reduced JNK phosphorylation, suppressed NF-κB activation, and normalized Wnt/β-catenin signaling, should be interpreted as coordinated redox-dependent responses rather than isolated events.^6^,^7^,^11^,^28^,^38^ Instead, they are the secondary consequences of stabilizing redox-sensitive signaling nodes. Based on this, we propose a Hierarchical Mechanistic Framework to delineate H_2_ action in OA:

(i) Primary intervention (chemical level): H_2_ directly scavenges ·OH and ONOO^−^. This is the root action, reducing the immediate chemical driving force of cellular injury.^23^,^27^

(ii) Secondary modulation (signaling level): The restoration of intracellular redox balance prevents the aberrant activation of redox-sensitive kinases (e.g., JNK) and transcription factors (e.g., NF-κB and β-catenin), thereby correcting the signaling network.^6^,^7^,^11^,^28^,^38^

(iii) Integrated outcomes (tissue level): Together, these upstream redox-stabilizing effects can attenuate regulated cell death (e.g., apoptosis and autophagy), dampen sterile inflammation, and support restoration of ECM homeostasis.^29^,^30^,^32^,^34^

This hierarchical view clarifies the central translational bottleneck. If H_2_ functions as a redox buffer that is rapidly consumed in oxidative environments, efficacy will depend on maintaining an adequate concentration–time profile at the site of pathology.^24^,^26^ This shifts the research focus from demonstrating what H_2_ can do to engineering how it is delivered, motivating the emphasis on exposure-controlled delivery systems in the next section.^26^

## Studies of Hydrogen Therapy in Osteoarthritis

Building on the mechanistic framework established, evidence for H_2_ therapy has expanded from cellular validation to preclinical models and preliminary clinical trials.^29^,^30^,^32^,^35^ Across these levels, a unifying principle emerges: therapeutic efficacy is strictly governed by the ability to maintain an effective concentration–time profile within the joint.^24^,^26^ Accordingly, this section synthesizes the evidence to emphasize a key translational constraint: biological outcomes depend on engineered joint exposure. To orient readers across evidence tiers, the OA-specific core studies identified in our search are summarized as an evidence map (**Figure 3**).

**Figure 3:**
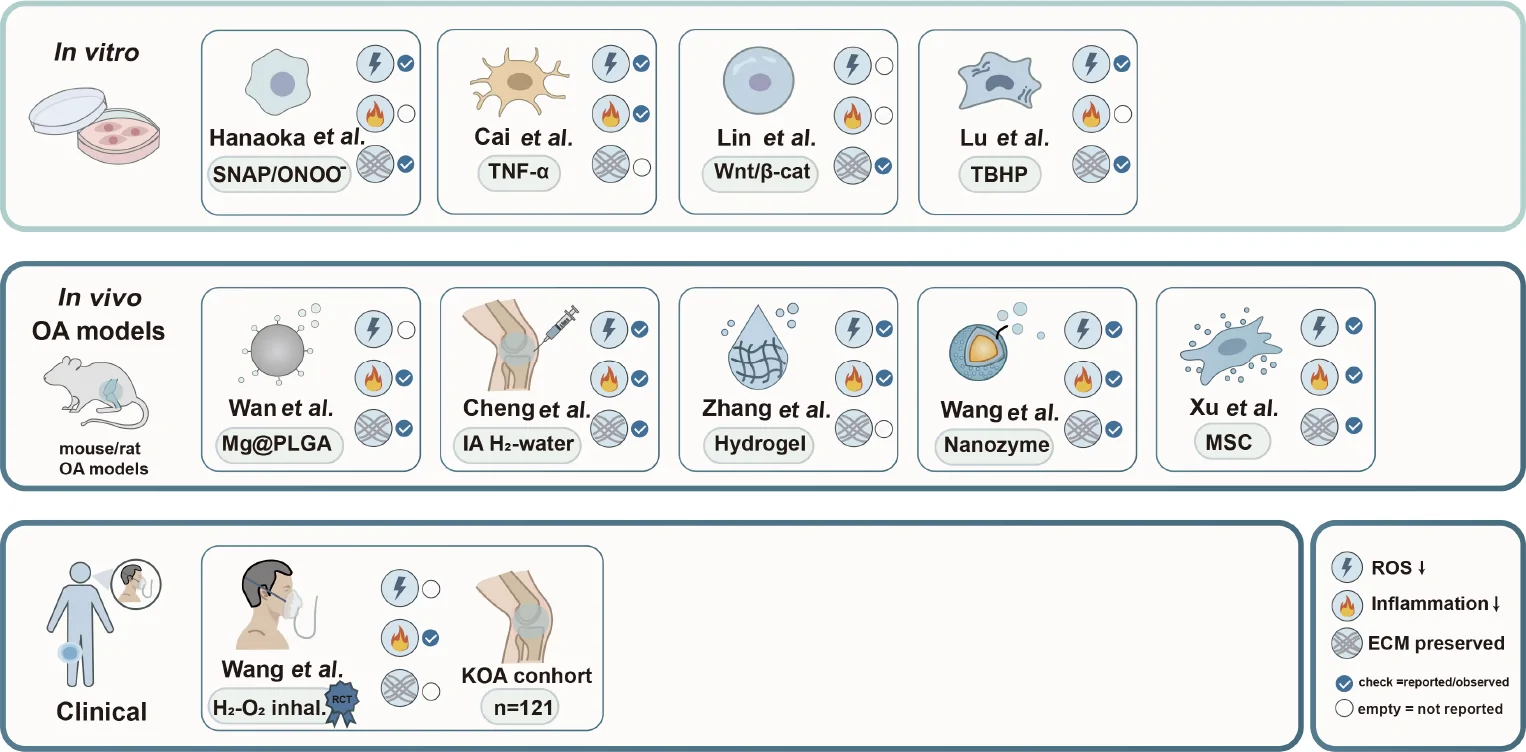
Evidence map of H_2_ therapy in OA. ECM: Extracellular matrix; H_2_: molecular hydrogen; IA: intra-articular; KOA: knee osteoarthritis; MSC: mesenchymal stem cell; OA: osteoarthritis; RCT: randomized controlled trial; ROS: reactive oxygen species.

### Chondroprotective effects at the cellular level

Cell models offer the clearest demonstration of the pharmacodynamic footprint of H_2_ when target cells are exposed to a defined dose.^21^ Under oxidant or cytokine challenge, chondrocytes show oxidative/nitrative injury, increased apoptosis, and a catabolic shift marked by higher matrix-degrading enzymes and reduced anabolic markers.^8^,^9^,^27^,^31^ Across these paradigms, H_2_ exposure tends to produce a coupled response: reduced nitrative and oxidative damage, improved viability, and partial restoration of ECM homeostasis.^27^,^30^,^31^

Mechanistically, the cellular literature converges on network modulation through redox-sensitive hubs rather than single-pathway blockade.^22^,^26^ The stress-kinase JNK axis repeatedly emerges as a link between oxidative stress, mitochondrial apoptosis, and catabolic transcription. Correspondingly, H_2_ treatment is often associated with reduced JNK activation and lower apoptotic signaling.^31^ H_2_ has also been linked to restraint of aberrant Wnt/β-catenin activity, helping preserve Sox9-related chondrocyte identity in models where Wnt activation is maladaptive.^28^

Two limitations should be considered when interpreting the findings. First, most *in vitro* studies are acute and reductionist, using bolus oxidants or single cytokines, which only partially represent chronic OA and multicellular crosstalk. Second, exposure is often described qualitatively rather than as a concentration–time metric.^24^ To address these gaps, future work should prioritize exposure-controlled pharmacodynamics and more physiological models, including chondrocyte–synoviocyte–macrophage co-cultures, OA synovial-fluid–conditioned systems, diffusion-controlled, and three-dimensional cartilage constructs.^49^,^50^,^51^ Such systems would allow the mapping of concentration–time profiles to specific pathway modulations and phenotypic outcomes, providing the pharmacokinetic/pharmacodynamic foundation needed to rationally design and compare delivery platforms *in vivo*. These *in vitro* insights highlight the need for optimized delivery systems, which are further validated in animal models of OA.

### Animal studies: bridging efficacy with delivery engineering

*In vivo* models validate whether the cellular footprint can be reproduced in an intact joint where diffusion, clearance, and tissue barriers dominate outcome.^24^,^49^,^50^,^51^ Animal studies consistently suggest that H_2_ is exposure-limited: transient systemic delivery or short-lived local boluses dissipate quickly and may not sustain target engagement over OA-relevant timescales. As a result, preclinical research increasingly integrates H_2_ with delivery engineering. The key comparative question is which approach can safely maintain intra-articular exposure.^25^,^26^

Current strategies can be summarized by how they engineer the joint concentration–time profile. Conceptually, engineered platforms reshape the intra-articular concentration–time profile of H_2_ from a rapid-decay bolus to a sustained exposure pattern (**Figure 4**).

**Figure 4:**
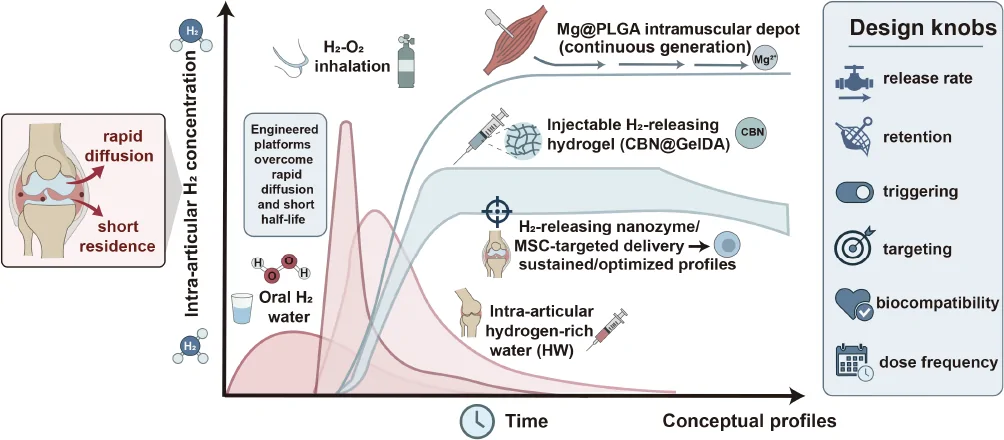
Exposure-limited pharmacokinetics of H_2_ in the joint and delivery-dependent reshaping of concentration–time profiles in OA. CBN: CaB_6_ nanosheets; GelDA: gelatin methacryloyl; H_2_: molecular hydrogen; HW: hydrogen-rich water; IA: intra-articular; OA: osteoarthritis; PK: pharmacokinetics.

#### Sustained-generation injectable hydrogels

Sustained-generation or depot systems aim to transform a transient gas into a prolonged intra-articular presence. By embedding an in situ H_2_ source whose reaction kinetics are diffusion- and microenvironment-limited, they extend effective exposure from minutes to days.

These platforms act as hydrated, pH-buffered microreactors that localize H_2_-generating components.^26^ For example, a calcium-boride-nanosheet-loaded hydrogel maintained slow H_2_ evolution for over 1 week in a rat OA model, effectively scavenging ROS and promoting reparative M2 macrophage polarization.^32^

#### Sustained-generation microparticle depots

These function as long-term reservoirs governed by magnesium hydrolysis (Mg + 2H_2_O → Mg(OH)_2_ + H_2_).^26^ Release is governed by a “passivation/activation cycle,” in which Mg(OH)_2_ surface films transiently slow hydrolysis until joint fluids disrupt the film and re-expose fresh Mg. Magnesium-poly(lactic-co-glycolic acid) (Mg@PLGA) microparticles leverage this mechanism to provide continuous H_2_ evolution, alleviating synovitis and cartilage destruction.^29^

#### Sustained-generation catalytic nanozymes

Platforms, such as self-releasing nanozymes (e.g., Se-HMPB@AB@COS), continuously generate H_2_ from chemically stored precursors (e.g., ammonia borane). These systems facilitate gradual, often pH-favored reactions within the OA microenvironment, simultaneously coupling delivery with mitochondrial quality control and antioxidant functions to reverse the chondrocyte senescence phenotype.^34^

#### Alternating magnetic field-enhanced programmable hydrogen generation systems

Alternating magnetic field (AMF)-enhanced H_2_ generation represents a prototypical “programmable” exposure-control strategy. While primarily developed for managing implant-related osteomyelitis, this work provides a proof-of-concept template for shifting H_2_ delivery from passive sustained release to externally modulatable dosing.^52^ In these platforms, magnesium implants exploit an AMF-induced eddy–thermal effect, in which localized heating accelerates Mg reactivity and thereby increases local generation of H_2_ gas together with co-released reaction products (e.g., Mg^2+^ and OH^−^).^52^,^53^ Notably, the reported therapeutic benefit in the osteomyelitis setting reflects a multifactorial mechanism (e.g., ROS scavenging by H_2_, microenvironmental proton depletion/alkalinization, antibacterial effects, and Mg^2+^-supported tissue regeneration) and has not been validated in OA or intra-articular applications.^52^

Conceptually, if adapted to an intra-articular depot, AMF triggering could address two translational needs simultaneously: (a) achieving sufficient local exposure despite rapid diffusion/clearance, and (b) enabling temporal control (e.g., on-demand pulsatile generation) to potentially align dosing with time-varying inflammatory activity without repeated injections.^54^,^55^,^56^ However, OA-specific translation would require rigorous calibration and safety validation. Unlike bacterial infection control, where alkalinization/proton depletion can be desirable, the delicate synovial environment necessitates strict monitoring of temperature rise, pH shifts, and ion/byproduct profiles.^52^,^57^,^58^ Future studies should therefore (a) define safe AMF parameters (e.g., frequency, field strength, and duty cycle) and establish joint-specific thermal maps, (b) quantify intra-articular H_2_ concentration–time profiles in synovial fluid and cartilage under repeated triggering, and (c) evaluate joint biocompatibility of co-generated byproducts and tissue responses, establishing AMF responsiveness, if feasible, as a verifiable exposure-control advantage for joint preservation rather than a purely conceptual feature.^24^,^29^,^32^,^59^,^60^ Together with passive depots, such externally triggered approaches further emphasize that controlling the concentration–time profile, rather than delivery route alone, may be the critical determinant of efficacy.^29^,^32^,^54^

#### Cell-enabled and combination platforms (“Hydrogen+”)

Cell-Enabled and Combination Platforms integrate H_2_ with regenerative or immunomodulatory interventions in a “Hydrogen+” format. The most advanced example is MSCs functionalized with H_2_-releasing nanoparticles. In this design, H_2_ is released in situ within the acidic OA microenvironment, protecting transplanted MSCs from oxidative stress and enhancing their immunomodulatory function (e.g., promoting M2 polarization). This combination has demonstrated superior cartilage regeneration in severe OA models.^33^

Across platforms, the conclusion is that structural and inflammatory improvements in animal OA models track more consistently with prolonged and localized exposure than with transient administration.^29^,^32^,^33^,^34^ This supports treating joint pharmacokinetics as a core experimental variable, not a background detail. Future *in vivo* work should report joint-level exposure measurements where feasible and link exposure to pathway modulation and structural endpoints. It should also evaluate biocompatibility and potential byproducts, including local pH or ion shifts.

### Clinical studies: status and next steps

Building on the mechanistic evidence and delivery constraints identified in preclinical models, clinical translation should be interpreted through the central variable of joint exposure. Accordingly, this section synthesizes the limited human evidence and reframes early outcomes within an exposure-designed framework to motivate exposure-verified next steps.

#### The translational gap: a pharmacokinetic mismatch

Despite the mechanistic clarity achieved in preclinical models, clinical translation remains in its infancy. High-quality human evidence is currently scarce, a deficit that likely reflects a fundamental technological bottleneck: the lack of clinically viable delivery systems capable of sustaining joint exposure. The existing evidence base is dominated by a single randomized controlled trial using systemic H_2_–oxygen inhalation, which showed only limited and transient symptomatic benefit; evidence for structural modification remains unavailable.^35^

#### Reinterpreting “negative” findings

Within the exposure-designed framework proposed in this review, such outcomes should not be interpreted as a failure of the mechanistic potential of H_2_, but rather as a confirmation of a pharmacokinetic mismatch. Systemic administration (e.g., inhalation or oral intake) is unlikely to produce a sustained, quantifiable concentration–time profile in synovial fluid. Such exposure is likely required to engage redox-sensitive pathways at the avascular cartilage interface.^24^ The “failure” to alter disease progression in early trials is, therefore, an expected consequence of insufficient local target engagement rather than a lack of biological efficacy.^24^,^35^

#### Strategic pivot: from administration to exposure-verified precision

To bridge this gap, future clinical research must evolve from empirical “administration trials” to rigorous “exposure-response studies.”^24^,^26^ This strategic pivot demands a unified focus on three interconnected domains.

(i) Route optimization: Clinical trials should shift from systemic routes toward local, exposure-optimized strategies (e.g., intra-articular depots).^17^,^29^,^32^ Demonstrating that a platform can maintain therapeutic levels in the human joint is a prerequisite for interpreting efficacy outcomes.^24^,^50^

(ii) Objective structure-function endpoints: To validate disease modification, endpoints must extend beyond subjective pain scores (which are prone to placebo effects) to include objective structural metrics.^61^,^62^ Quantitative magnetic resonance imaging measures (e.g., cartilage thickness/volume and bone marrow lesions) together with validated synovial biomarker panels are critical for distinguishing true biological modification from transient symptom fluctuation.^63^,^64^

(iii) Biomarker-guided stratification: Given the heterogeneity of OA, H_2_ is unlikely to benefit all patients equally.^1^,^65^ Future designs should incorporate phenotype stratification, selecting patients with elevated baseline oxidative stress or inflammatory signatures.^64^,^65^ This precision medicine approach ensures that the therapy is targeted toward the pathophysiology it is best designed to treat.

#### Overall synthesis

Overall, a coherent translational logic emerges across evidence tiers. Cellular studies define redox-sensitive targets, animal models show that efficacy depends on engineered retention, and early clinical experience suggests systemic exposure is insufficient for local disease modification.^24^,^28^,^29^,^31^,^32^,^33^,^34^,^35^ The field is at a decisive juncture: clinical success will require treating joint pharmacokinetics as a primary design variable rather than a background detail. Accordingly, local exposure should be verified and linked to objective structural outcomes to translate the multi-target promises of H_2_ into lasting clinical benefit for OA.^63^,^64^

## Toward an Integrated Translational Roadmap: from Hydrogen Administration to Exposure-Designed Therapy

To bridge the gap between preclinical promise and clinical reality identified, the field must undergo a paradigm shift: moving from simple “hydrogen administration” to exposure-designed H_2_ therapy.^24^,^26^ The literature suggests that efficacy is not merely a question of biology, but of physics, specifically, the challenge of maintaining therapeutic residence in a highly diffusive joint environment.^50^ Therefore, successful translation will require an integrated roadmap that co-develops exposure-designed delivery systems, rational “Hydrogen+” combination strategies, and biomarker-guided patient stratification.^33^,^65^ From a practical standpoint, existing OA-oriented H_2_ delivery approaches can be grouped into five categories with distinct implementation trade-offs (**Figure 5**).

**Figure 5:**
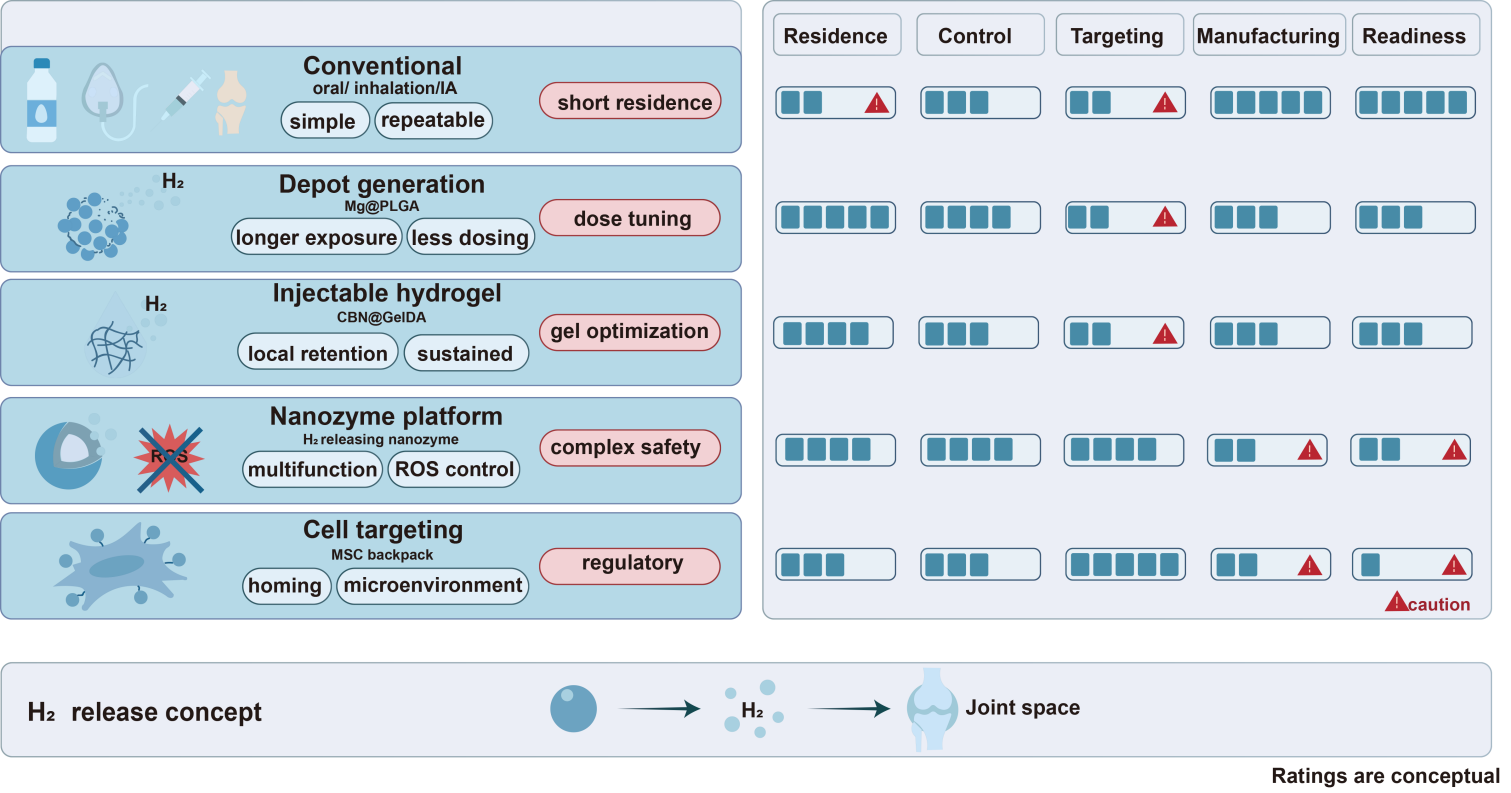
Taxonomy of H_2_ delivery platforms for OA and qualitative translational trade-offs. H_2_: Molecular hydrogen; MSC: mesenchymal stem cell; OA: osteoarthritis; ROS: reactive oxygen species.

### Engineering intelligent, joint-adaptive delivery platforms

Mechanistic plausibility is now well supported; however, translation will depend on sustaining meaningful intra-articular exposure. Therefore, future material design must transcend passive carriers and function as active controllers of the joint concentration–time profile. Next-generation platforms should be benchmarked against rigorous pharmacokinetic standards. It is no longer sufficient to claim “sustained release”; materials should be characterized by H_2_ generation kinetics, total H_2_ yield, and spatial distribution within the synovial cavity.^24^,^25^,^26^ Ideal systems should be “joint-adaptive,” leveraging pathological cues (e.g., acidic pH, elevated ROS, or mechanical loading) to trigger feedback-regulated release.^25^,^34^ Crucially, the biocompatibility of these systems must be evaluated in the context of their byproducts. Local consequences of carrier degradation, such as pH shifts from magnesium hydrolysis or ion release, should be quantified to ensure the vehicle does not aggravate joint inflammation.^25^,^29^

### Rationalizing “Hydrogen+” strategies: microenvironment conditioning

Once exposure can be reliably controlled, the next challenge is to maximize benefit across heterogeneous OA phenotypes.^65^ In this context, H_2_ is unlikely to serve as a universal monotherapy for end-stage disease and may be better positioned as a foundational microenvironment conditioner.^26^,^65^

By lowering oxidative and inflammatory barriers that limit repair, H_2_ may potentiate companion interventions and improve their effectiveness.^33^ This supports “Hydrogen^+^” strategies, where H_2_ is combined with regenerative modalities (e.g., MSCs, exosomes) or intra-articular biologics to improve their survival and bioactivity.^18^,^33^,^66^ Examples include H_2_ + MSCs (to mitigate oxidative senescence and improve engraftment effects), H_2_ + MSC-derived exosomes (to preserve bioactivity in ROS-rich joints), H_2_ + intra-articular biologics/anti-inflammatory agents (to reduce inflammatory barriers), and co-delivery platforms (hydrogels) that synchronize local retention of both H_2_ and regenerative cues.

These combinations must not be assembled opportunistically. They require hypothesis-driven designs (e.g., using factorial trials) to isolate the contribution of each component.^67^ For instance, demonstrating that H_2_ specifically prevents oxidative senescence in transplanted cells would provide a mechanistic rationale for its inclusion in regenerative regimens.^33^,^34^,^68^

### From aspiration to operation: biomarker-guided stratification

Even with optimized delivery and rational “Hydrogen^+^” combinations, benefit will not be uniform across patients, so responder identification and target engagement must be built into the strategy.^65^,^69^ Therefore, precision medicine in OA must transition from a general aspiration to a testable operational approach.

Clinical evaluation should utilize a linked biomarker panel spanning (i) redox indices (to verify target engagement), (ii) synovitis metrics (to assess anti-inflammatory potency), and (iii) cartilage turnover markers (to track structural modification).^68^,^69^,^70^ Matching these profiles to specific delivery modalities ensures that the therapy is targeted toward the biological mechanism it is best designed to modulate.^25^,^65^

Taken together, delivery optimization, rational combinations, and biomarker-guided stratification form a single translational chain from engineered exposure to measurable biological outcomes. Accordingly, the next phase of H_2_ research should be structured around explicit exposure–mechanism–outcome links. Specifically, translation hinges on three linked elements: engineering to achieve adequate intra-articular exposure, biomarkers to confirm target engagement, and stratification to match therapy to the phenotypes most likely to respond.^26^,^65^,^69^ This integrated program offers the most viable route to converting the multi-target potential of molecular H_2_ into an actionable, disease-modifying strategy for OA. These priorities define an exposure-informed translational roadmap for advancing H_2_ toward disease modification in OA (**Figure 6**).

**Figure 6:**
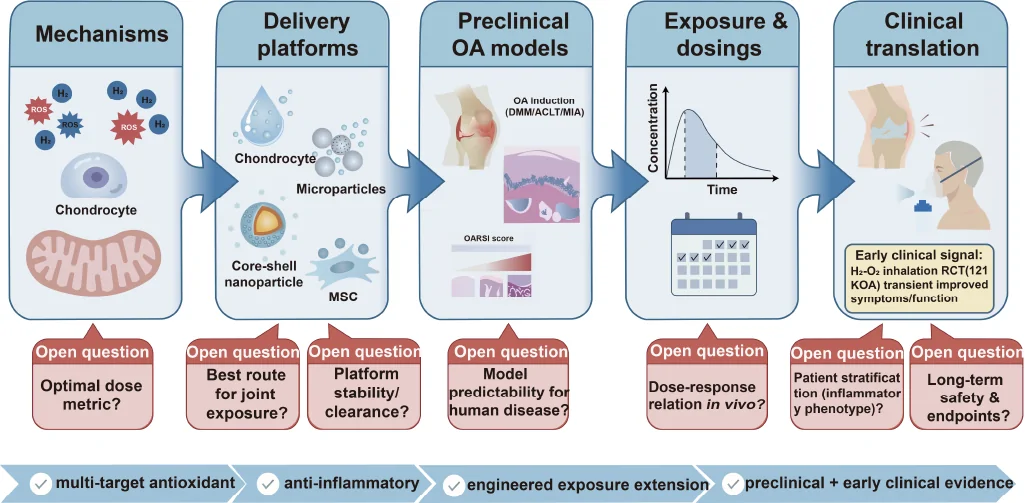
Translational roadmap and key open questions for H_2_ therapy in OA. H_2_: Molecular hydrogen; MRI: magnetic resonance imaging; OA: osteoarthritis; OARSI: Osteoarthritis Research Society International; RCT: randomized controlled trial.s

## Limitations

This review has several methodological limitations. First, the existing body of evidence exhibits substantial heterogeneity across OA models, H_2_ administration routes, dosing regimens, and outcome assessments, thereby constraining the validity and generalizability of cross-study comparisons. Second, many mechanistic inferences are derived primarily from preclinical or in vitro investigations; robust clinical evidence substantiating causal relationships between H_2_ intervention and structural disease modification in human OA remains limited. Third, pharmacokinetic data characterizing intra-articular H_2_ exposure are sparse and inconsistently reported, while engineered delivery systems vary considerably in design rationale, safety profiling, and readiness for clinical translation. Finally, as a narrative synthesis, not a systematic review, the present analysis did not adhere to a prespecified protocol for study identification, selection, or quality appraisal, nor did it incorporate quantitative synthesis (e.g., meta-analysis); consequently, it may be susceptible to selection bias, publication bias, and other forms of systematic error.

## Conclusion

H_2_ is a distinctive therapeutic candidate for OA, as it quenches cytotoxic radicals and modulates downstream redox-sensitive networks governing inflammation, regulated cell death, and matrix metabolism.^21^,^23^,^71^ This multi-target, “network-level” mechanism aligns precisely with the multifactorial nature of OA.^65^ While mechanistic plausibility is well-established, the primary translational challenge lies in ensuring controlled delivery that maintains effective intra-articular exposure.^25^,^26^ Early clinical trials have underscored this pharmacokinetic hurdle, revealing a critical disconnect between route of delivery and sustained target engagement.^35^

Therefore, the path forward requires a dual, integrated focus. First, exposure-designed delivery platforms should be paired with joint-level pharmacokinetic/pharmacodynamic validation to define exposure–response relationships and confirm local biocompatibility. Second, clinical evaluation should advance through trials that include objective structural endpoints, biomarker-verified target engagement, and patient stratification based on redox or inflammatory phenotypes.^65^,^69^,^70^,^72^ By anchoring its development in this exposure-informed and biomarker-linked framework, molecular H_2_ has the potential to transition from a compelling preclinical agent to a clinically validated DMOAD for OA.

## Additional file:

***Additional file 1:*** *Systematic literature search strategy and study selection.*

This file describes the predefined search strategy, eligibility criteria, and screening process used to identify primary studies investigating molecular hydrogen (H_2_) therapy in osteoarthritis (OA).

**S1. Databases and search period**

• Databases: PubMed, Web of Science Core Collection, and Google Scholar.

• Time window: January 1, 2005, through October 8, 2025.

• Language: English.

• Study type filter: primary research only (*in vitro*, *in vivo*, or clinical studies).

**S2. Search strategy**

The search strategy integrated free-text keywords with controlled vocabulary where applicable (Additional Table 1). References related to hydrogen sulfide (H_2_S) were excluded to avoid conflation with other medical gases. In addition to database searching, citation tracking and manual screening of reference lists from eligible articles and recent reviews were performed to ensure comprehensiveness.

**Additional Table 1:** Search query

Database	Search query (as executed)	Notes
PubMed	(("molecular hydrogen" OR "hydrogen gas" OR H2 OR "hydrogen-rich water") AND ("osteoarthritis" OR OA OR chondrocyte)) NOT ("hydrogen sulfide" OR H2S)	Keywords and MeSH mapping were allowed by PubMed automatically; results were limited to the time window above.
Web of Science Core Collection	TS=("molecular hydrogen" OR "hydrogen gas" OR H2 OR "hydrogen-rich water") AND TS=("osteoarthritis" OR OA OR chondrocyte) NOT TS=("hydrogen sulfide" OR H2S)	Topic (TS) search across title/abstract/keywords; results limited to the time window above.
Google Scholar	("molecular hydrogen" OR "hydrogen gas" OR H2 OR "hydrogen-rich water") ("osteoarthritis" OR OA OR chondrocyte) -("hydrogen sulfide" OR H2S)	Multiple keyword combinations were used; results were screened by relevance (sorted by Google Scholar default relevance).

**S3. Eligibility criteria**

Inclusion criteria:

• Original primary studies (cell/*in vitro*, animal/*in vivo*, or clinical) that evaluated an H_2_-based intervention in the context of OA.

• OA-relevant models or populations (e.g., IL-1β/TNF-α challenged chondrocytes; surgically induced OA models such as DMM or Hulth-type procedures; human OA cohorts).

• Interventions delivering molecular hydrogen, including but not limited to hydrogen-rich water/saline, inhaled H_2_/H_2_–O_2_ mixtures, hydrogen-generating/depot biomaterials, or hydrogen-releasing nanozyme platforms.

• Reported OA-relevant outcomes (e.g., pain/function scores, cartilage histology, synovitis, oxidative/inflammatory biomarkers, ECM anabolic/catabolic markers).

Exclusion criteria:

• Non-English publications.

• Non-primary publications (reviews, editorials, commentaries, letters without original data).

• Studies not related to OA (e.g., other arthritides or non-joint indications).

• Studies focused on other medical gases or pathways without molecular hydrogen as the intervention of interest (including hydrogen sulfide/H_2_S).

• Duplicate records (the most complete report was retained).

**S4. Screening and selection process**

Two reviewers independently screened titles and abstracts, followed by full-text review for potentially eligible studies, using the criteria above. Disagreements were resolved through discussion until consensus was reached. Manual citation tracking (both backward and forward) was performed for the included studies to identify additional eligible primary articles.

**S5. Definition of the core evidence set**

Using the screening process above, we identified 10 core studies directly investigating hydrogen therapy in OA (nine preclinical studies and one clinical trial). These core studies form the basis of the evidence synthesis presented in Table 1. Additional references cited in the main text for background (OA pathophysiology, general biomaterials/delivery technologies, trial methodology) were used for contextual purposes and were not counted as part of this core evidence set.

Core studies included:

• Hanaoka et al.^27^ — preclinical

• Cai et al.^6^ — preclinical

• Lin et al.^28^ — preclinical

• Wan et al.^29^ — preclinical

• Cheng et al.^30^ — preclinical

• Lu et al.^31^ — preclinical

• Zhang et al.^32^ — preclinical

• Wang et al.^34^ — preclinical

• Xu et al.^33^ — preclinical

• Wang et al.^35^ — clinical randomized controlled trial (hydrogen–oxygen inhalation adjunct to home-based exercise)

**S6. Data extraction and synthesis (summary)**

• For each core study, we extracted: study design/model, hydrogen delivery modality, dosing/exposure information when available, key molecular/pathway readouts, and OA-relevant phenotypic outcomes.

• Evidence was synthesized narratively with emphasis on mechanistic pathways (Section 2) and exposure-limited translation/delivery engineering (Sections 3 and 4).

**Facts**

1. Osteoarthritis (OA) is multifactorial, involving oxidative stress, inflammation, chondrocyte damage, and death. Multi-target interventions are rational.
2. Molecular hydrogen (H_2_) protects against OA via redox modulation, reduced inflammation, and improved joint immune microenvironment, benefiting cartilage.
3. Limited intra-articular H_2_ exposure, rapid diffusion, and short residence time are key clinical barriers. Pharmacokinetics and exposure control are critical.
4. Engineered delivery strategies prolong intra-articular H_2_ availability, showing chondroprotection in OA models.
5. Clinical evidence is preliminary, focusing on symptom relief. Future trials need optimized H_2_ delivery, standardized structural endpoints, and biomarker stratification.

**Open Questions**

1. What intra-articular H_2_ exposure metrics/profiles suit OA stages? How to define/quantify “disease modification”?
2. How to evaluate long-term biocompatibility/adverse effects of sustained H_2_ systems (e.g., Mg depots, nano-materials)?
3. Which biomarkers can stratify patients, predict H_2_ therapy response, and monitor efficacy in precision medicine?
4. Can H_2_ synergize with stem cells and extracellular vesicles for joint repair? What are the molecular/cellular mechanisms?
5. How can we design trials to objectively assess the disease-modifying effects of H_2_? How can we integrate multimodal endpoints, biomarkers, and patient outcomes, while ensuring adequate follow-up?

## Data Availability

*All data generated or analyzed in this study are included in this published article and its additional file.*
